# Engineering Lignin‐Based Tubular Hydrogel Scaffolds for Load‐Bearing Biomedical Applications

**DOI:** 10.1002/cssc.202501520

**Published:** 2025-09-11

**Authors:** Muhammad Muddasar, Grace Joyce, Mathilde Pouzier, Aleksandra Serafin, Maurice N. Collins

**Affiliations:** ^1^ Stokes Laboratories School of Engineering Bernal Institute University of Limerick Limerick V94 T9PX Ireland; ^2^ Advanced Materials and Bioengineering Research (AMBER) Centre University of Limerick V94 T9PX Ireland

**Keywords:** lignin, lignin‐based hydrogels, soft tissue engineering, sustainable biomaterials, tubular biomaterials

## Abstract

The development of mechanically robust, biocompatible, and biodegradable hydrogels remains a significant challenge for biomedical applications involving load‐bearing soft tissues. Herein, a tubular lignin‐derived hydrogel is engineered to assess its physicochemical, mechanical, and biological properties. Kraft and organosolv lignin are systematically compared at varying crosslinker concentrations to determine their effect on pore morphology, swelling behavior, and mechanical performance. Organosolv lignin formulations at 5% crosslinker concentration demonstrate an optimal balance between strength (ultimate tensile strength: 83.14 ± 0.16 kPa), flexibility (elongation: up to 176%), and hydration (swelling capacity: 261%), and are further fabricated into tubular geometries, with and without polypropylene mesh reinforcement. The reinforced tubular constructs exhibit superior mechanical strength, sustained performance over 100 fatigue cycles, and cytocompatibility with fibroblast cultures (cell viability: 85.5–86.5% after 96 h). These findings highlight the potential of lignin‐based hydrogel scaffolds as sustainable, tunable platforms for a broad range of biomedical applications requiring soft, mechanically resilient, and tubular structures, such as tendon repair, vascular conduits, and nerve regeneration.

## Graphical abstract

Flexible tubular shaped lignin based hydrogels.

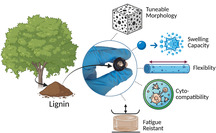

## 1 Introduction

Soft tissue injuries, including those affecting tendons, ligaments, and joint capsules, account for 45% of the 33 million musculoskeletal injuries recorded annually in the United States (US).^[^ ^1^ ^]^ The combined annual expenditure on these injuries in the United States exceeds 390 billion USD.^[^ ^2^ ^]^ While tendon injuries are notably prevalent, with surgical interventions often required for Achilles tendons (18 per 100,000), rotator cuffs (131 per 100,000), and flexor tendons (33.2 per 100,000), similar challenges exist in other load‐bearing soft tissues, such as ligaments, blood vessels, and peripheral nerves.^[^ ^3^ ^]^ These injuries place a heavy burden on healthcare systems due to surgery, rehabilitation, and long‐term recovery needs. Furthermore, the incidence of individuals affected by these injuries is anticipated to rise due to the ongoing increase in life expectancy and the growing participation in athletic activities.^[^ ^4^ ^]^

The healing mechanisms of soft connective tissues like tendons and ligaments are complex and often result in incomplete functional recovery. Traditional treatments include conservative management, surgical repair (e.g., sutures, autografts, allografts, and xenografts), and bioengineering strategies such as cell therapy, growth factor delivery, and gene editing.^[^ ^5^ ^]^ However, many approaches yield inconsistent or suboptimal outcomes, often leading to reinjury or impaired quality of life.^[^ ^6^ ^]^ Thus, improved materials and scaffold designs are needed to better support healing in these mechanically demanding environments.

Hydrogels have gained significant attention in tissue engineering due to their unique ability to mimic the native extracellular matrix (ECM) by providing a hydrated, 3D environment conducive to cell proliferation and differentiation.^[^ ^7^, ^8^ ^]^ Their tunable mechanical properties and high‐water content make them particularly suitable for soft tissue engineering, where replicating the complex biomechanical and biological environment is critical. However, a major limitation of conventional hydrogels lies in their insufficient mechanical strength and durability, which restrict their use in load‐bearing tissues that are subjected to repetitive tensile forces.^[^ ^9^ ^]^ Consequently, there is a pressing need for advanced hydrogel scaffolds that combine mechanical robustness, biological functionality, and long‐term durability in mechanically demanding environments.

Lignin, an abundant and renewable biopolymer sourced from lignocellulosic biomass, offers a sustainable and eco‐friendly alternative to traditional synthetic biomaterials. Its natural antioxidant, biodegradable, and biocompatible properties not only enhance hydrogel performance but also align with the growing demand for environmentally responsible biomaterials.^[^ ^10^, ^11^, ^12^, ^13^ ^]^ Incorporating lignin into hydrogels can improve mechanical strength and stability, making them more suitable for the dynamic, high‐stress environment of soft tissue engineering.^[^ ^14^ ^]^ Recent efforts to improve lignin‐based hydrogels for biomedical applications have focused on chemical modification techniques, such as methacrylation, thiolation, and lignin‐polymer composite formulations, to enhance crosslinking density, mechanical strength, and degradation control.^[^ ^15^, ^16^, ^17^ ^]^ While these strategies have demonstrated potential in static or quasistatic conditions, there is a significant lack of fundamental studies investigating how lignin type and crosslinker concentration influence the mechanical resilience, flexibility, and long‐term durability of lignin‐based hydrogels under dynamic mechanical loading, such as creep and fatigue. These dynamic loading modes are highly relevant to load‐bearing tissues like tendons and ligaments, yet remain underexplored.

Moreover, while significant progress has been made in enhancing the chemical and physical properties of lignin‐based hydrogels, their fabrication into tubular structures, crucial for replicating the natural architecture of tendons, blood vessels, and nerves, remains underexplored. Tubular scaffolds offer clear advantages by better mimicking native tissue geometry, which supports improved cell attachment, proliferation, and integration.^[^ ^18^ ^]^ While fabrication methods such as template‐assisted radical polymerization, molding techniques, and 3D‐printed tubular scaffold casting have enabled the development of tubular structures using elastomeric, gelatin‐based, or poly(vinyl alcohol) (PVA)‐based materials,^[^ ^19^, ^20^, ^21^ ^]^ to the best of our knowledge, none have successfully applied these approaches to lignin‐based hydrogels. As a result, the design, mechanical characterization, and structural evaluation of tubular lignin hydrogel remain underexplored. This knowledge gap hinders the development of lignin‐based hydrogels as viable biomedical devices, particularly for applications that demand precise structural design and sustained mechanical durability. Therefore, a unified strategy that integrates both dynamic mechanical performance and tubular architecture is essential to advance lignin hydrogel systems for practical use.

Herein, this study addresses these gaps for the first time and aims to systematically evaluate the influence of lignin type and crosslinker concentration on the chemical, mechanical, and structural properties of lignin‐based hydrogels, with a focus on their dynamic mechanical behavior, such as fatigue resistance, recoverability, and durability under cyclic loading. In addition, this research will undertake the fabrication and characterization of tubular lignin‐based hydrogel scaffolds, assessing their mechanical robustness, structural stability, and biocompatibility for tissue engineering applications. Through comprehensive chemical analyses, mechanical testing, and structural evaluations, this work seeks to identify optimal formulations and configurations that balance sustainability, mechanical performance, and biological functionality. Ultimately, this study aims to advance lignin‐based hydrogel technology toward scalable, high‐performance, and biodegradable scaffolds suited for tendon repair and other regenerative medicine applications where both form and function are critical.

## 2 Results and Discussion

**Figure 1:**
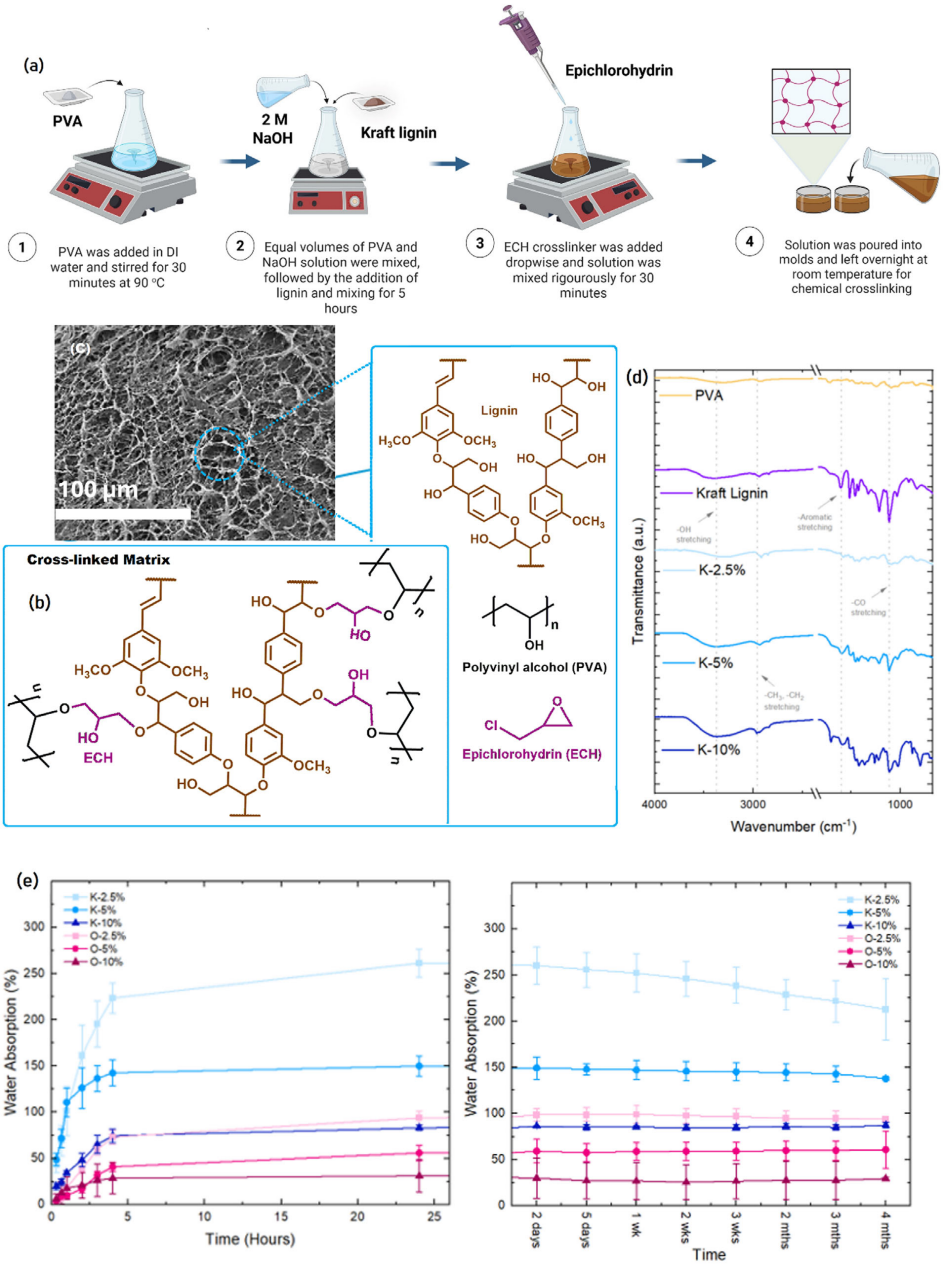
Lignin‐derived hydrogels: a) Schematics of synthesis protocol, b) crosslinking mechanism, c) SEM image showing hydrogel morphology, d) FTIR spectra of precursors and synthesized hydrogels, and e) Swelling capacity analysis.

**Figure** **1**a illustrates the synthesis procedure of lignin‐derived hydrogels. The proposed crosslinking mechanism of the lignin–PVA hydrogel system facilitated by epichlorohydrin (ECH) as a bifunctional crosslinking agent (Figure 1b). ECH contains a reactive epoxide ring adjacent to a chloromethyl group, with the epoxide carbon atoms being electrophilic due to the strong electronegativity of the ring oxygen, which withdraws electron density and renders the adjacent carbons susceptible to nucleophilic attack. Under alkaline conditions (NaOH), the hydroxyl groups on both PVA and lignin are deprotonated, forming alkoxide ions (RO^−^), which act as strong nucleophiles. The crosslinking reaction proceeds via a concerted SN2‐type nucleophilic substitution mechanism, wherein the alkoxide ions preferentially attack the less sterically hindered epoxide carbon of ECH.^[^ ^22^ ^]^ This leads to ring opening of the epoxide and displacement of the chloride ion as the leaving group. The result is covalent bond formation between lignin and PVA through ether linkages, effectively generating a crosslinked 3D polymeric network. This mechanism enhances the structural integrity of the hydrogel and contributes to its mechanical stability and chemical resistance under physiological conditions.

Scanning electron microscopy (SEM) analysis revealed that all lignin‐PVA hydrogels exhibited a 3D interconnected porous network formed via freeze‐drying and chemical crosslinking, as shown in Figure 1c and S2, Supporting Information. The incorporation of lignin significantly influenced the network morphology, leading to a bimodal pore size distribution consisting of mesopores (2–50 nm) and micropores (<2 nm). This structural variation is attributed to lignin dual role as a heterogeneous nucleating agent and an irregular crosslinking site, due to its variable phenolic and aliphatic hydroxyl content.^[^ ^23^, ^24^ ^]^ At low ECH concentration (2.5% v/v), hydrogels formed loosely crosslinked, open mesoporous structures, with organosolv iignin (OL) hydrogels showing more uniform pores due to its lower molecular weight and higher phenolic content than that of kraft lignin (KL) hydrogels. On the other end, the highest ECH concentration (10% v/v), both KL and OL hydrogels transitioned to microporous architectures with significantly reduced pore connectivity and increased rigidity. This shift reflects an over‐crosslinked regime, where excessive ECH limits polymer mobility and induces vitrification‐like behavior, potentially compromising swelling, mechanical flexibility, and biodegradability under physiological conditions.

Fourier transform infrared spectroscopy (FTIR) was employed to analyze the chemical structure and confirm successful crosslinking in lignin–PVA hydrogels (Figure 1d and S2, Supporting Information). Broad absorption bands observed in the 3500–3000 cm^−1^ region were attributed to O–H stretching vibrations, indicating the presence of both aliphatic and aromatic hydroxyl groups inherent to lignin and PVA.^[^ ^25^ ^]^ KL exhibited a distinct peak at 1740 cm^−1^, corresponding to C=O stretching vibrations of ester or carboxylic acid groups, whereas OL displayed a more intense band around 1700 cm^−1^, suggesting a higher concentration of carboxylic functionalities introduced during the organosolv process.^[^ ^26^ ^]^ C–H stretching vibrations appeared between 2840 and 3000 cm^−1^, while characteristic aromatic skeletal vibrations were identified in the region of 1595–1510 cm^−1^. Peaks associated with —CH_2_ and —CH_3_ bending modes were observed at 1460–1420 cm^−1^. These spectral features varied with lignin type and ECH concentration, reflecting compositional and structural differences in the hydrogels. Notably, the disappearance of epoxide‐related absorption bands (926–961 cm^−1^) and chlorinated species (750–780 cm^−1^) confirmed the successful reaction of ECH and the removal of unreacted crosslinker. Increasing ECH concentration led to a progressive reduction in O–H stretching intensity and the emergence of more prominent ether (C—O—C) bands around 1085 cm^−1^, indicating increased crosslinking density through covalent bond formation. Additionally, shifts in absorption bands at 1586, 1106, and 876 cm^−1^ were indicative of reduced PVA crystallinity, as a consequence of network formation.^[^ ^7^ ^]^

Swelling behavior of KL and OL lignin‐based hydrogels was investigated over a prolonged period in phosphate‐buffered saline (PBS) at 37 °C, with trends clearly influenced by crosslinker concentration and lignin type (Figure 1e). All hydrogels followed a typical swelling pattern, initial rapid uptake followed by plateauing at equilibrium within 24 h. KL‐based hydrogels consistently exhibited higher swelling capacities compared to OL‐based hydrogels, and this enhanced swelling behavior is attributed to the greater abundance of hydrophilic functional groups in KL, particularly phenolic hydroxyl groups, which are generated through cleavage of β‐O‐4 linkages during the alkaline conditions of the Kraft pulping process.^[^ ^27^ ^]^ Crosslinker concentration played a critical role in modulating swelling behavior. Hydrogels with 2.5% ECH showed the highest swelling (KL: ≈261%, OL: ≈99%) due to looser networks and larger pore sizes, whereas increasing ECH to 5% and 10% significantly reduced swelling due to tighter crosslinking and reduced porosity. This trend aligns with morphological findings. However, while lower crosslinking enhances swelling, it may compromise mechanical integrity, an essential consideration for biomedical applications involving load‐bearing soft tissues, which require a balance between swelling, degradation rate, and mechanical strength. Preliminary data suggest that O‐2.5% and K‐2.5% hydrogels may degrade faster due to lower crosslink density, though longer studies are needed for confirmation.

**Figure 2:**
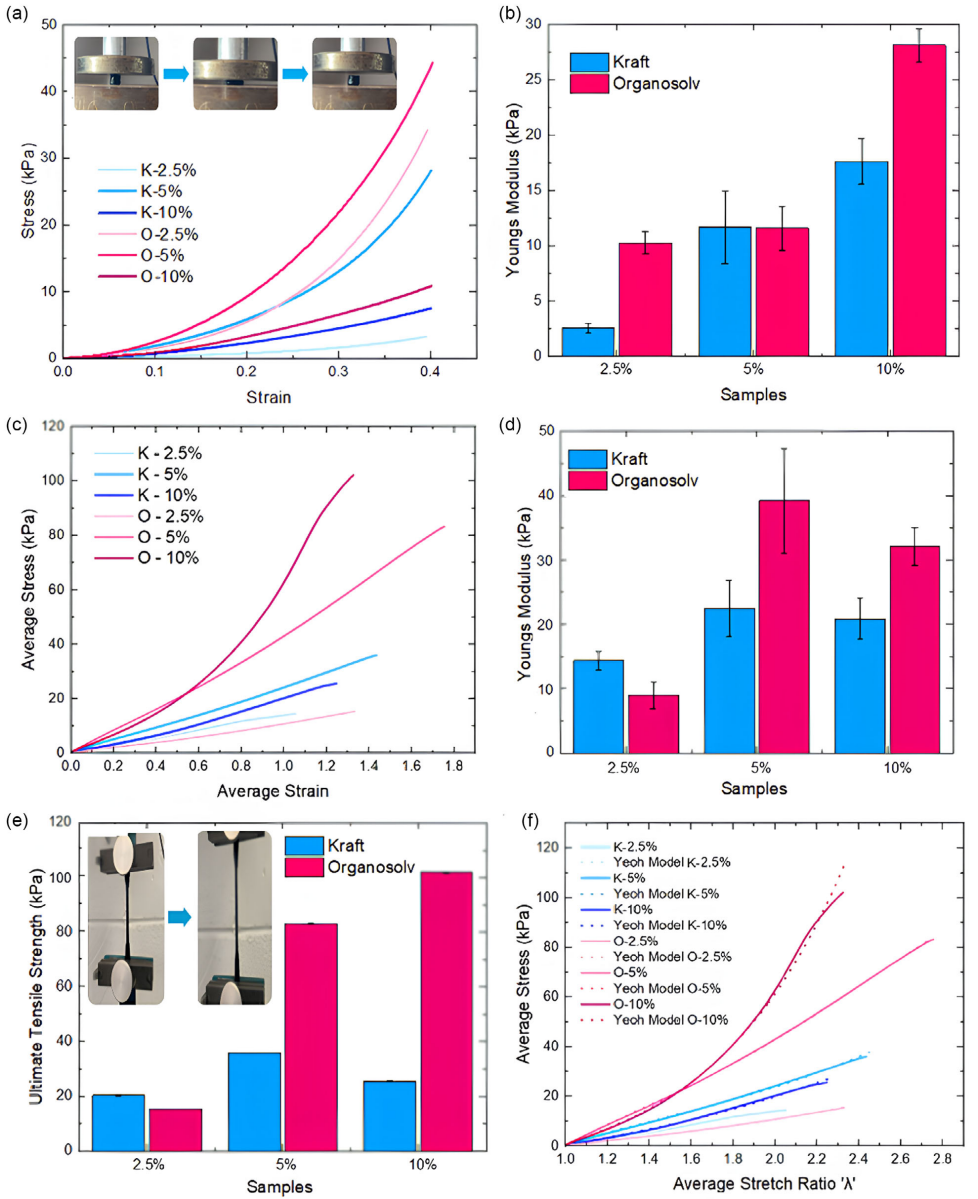
Mechanical testing of lignin‐derived hydrogels. a) Compression testing, with corresponding b) Young's modulus. c) Tensile testing and d) Young's modulus in tension. e) Ultimate tensile strength (UTS) of the hydrogels. f) Yeoh model fitting applied to the tensile data.

Uniaxial compression testing was conducted to evaluate the mechanical response of the synthesized lignin–PVA hydrogels. The compressive stress–strain behavior up to 40% strain is illustrated in **Figure** **2**a, with corresponding Young's modulus (E) values shown in Figure 1b. All measurements were performed using a 200 N load cell at a constant rate of 10 mm/min (n = 3, mean ± SD). The stress–strain curves revealed a nonlinear elastic behavior, typical of hydrogel materials, with stress increasing progressively with strain. Notably, increasing lignin content led to a steeper stress–strain slope and a range of Young's modulus (2.57 to 39 ± 8 kPa), indicating enhanced stiffness and load‐bearing capacity. This trend is attributed to the greater density of functional groups in lignin, particularly phenolic hydroxyls, which act as additional crosslinking sites for epoxide‐mediated reactions with ECH. As a result, a more compact and tightly crosslinked network is formed.^[^ ^28^ ^]^ This hypothesis is supported by SEM analysis (Figure 1b), which revealed decreased pore size and reduced porosity with increasing lignin content, consistent with a denser internal structure. The enhanced mechanical performance of high‐lignin‐content hydrogels underscores the role of lignin not only as a filler but also as a reactive structural component that contributes to the overall crosslinking density and network integrity.

While tendons have a Young's modulus closer to the GPa/MPa range naturally^[^ ^29^ ^]^ with many tissue‐engineered hydrogel systems also aiming for such stiffnesses, the application of hydrogels with stiffnesses in the kPa range also holds merit. After injury, healing tendons exhibit a stiffness of ≈30–200 kPa (depending on the location, type etc). The stiffness of the lignin hydrogels matching this value allows for the creation of a biomimetic environment that supports regeneration and the avoidance of a foreign body response or fibrotic scarring/encapsulation, which is so often the case with hydrogels with greater stiffness.^[^ ^30^ ^]^ Polyacrylamide substrates functionalized with whole length fibronectin and type‐I collagen with stiffnesses between 30–50 kPa have been shown to more effectively support the tenogenic behaviors of bone marrow stromal cells compared with softer or higher stiffness.^[^ ^31^ ^]^ The same results were observed by Sun et al., when tendon derived stem cells were cultured on gelatin hydrogels of various stiffness (≈2–20 kPa). Differentiation of the stem cells into tenogenic, chondrogenic, and osteogenic lineages were inhibited on stiff hydrogel, indicating that matrix stiffness modulated the proliferation and differentiation of the stem cells.^[^ ^32^ ^]^ However, other studies claimed that the inverse was true, with stem cell differentiation towards the tendon lineage being achieved on much stiffer hydrogels, thus pushing for more robust studies to be conducted in order to reputedly claim which hydrogel stiffness is ideal.^[^ ^30^ ^]^

Tensile testing was employed to assess the mechanical behavior of the hydrogels under conditions that simulate physiological tensile loading, particularly relevant for applications such as tendon and ligament scaffolds. All hydrogels displayed considerable extensibility, with failure strains exceeding 130% (Figure 1c), surpassing the strain threshold (≥8%–10%) for macroscopic failure in native tendons. OL‐based hydrogels demonstrated greater elongation (133%–176%) than KL‐based ones (135%–158%), indicating improved deformability due to OL's more flexible molecular architecture. Young's modulus (Figure 2d) and ultimate tensile strength (UTS) (Figure 2e) increased with crosslinker concentration, reflecting enhanced network density.

OL hydrogels consistently outperformed KL samples at equivalent concentrations. O‐5% reached the highest modulus (39 ± 8 kPa) and tensile strength (83.14 ± 0.16 kPa), while O‐10% further improved UTS to 102 ± 0.13 kPa. In contrast, KL samples achieved lower values across all metrics. These differences are attributed to the highly condensed and structurally heterogeneous nature of KL, resulting from the harsh conditions of the Kraft pulping process, which reduces its reactivity and limits effective crosslinking. In contrast, OL possesses a less condensed, more homogeneous structure with a higher content of reactive hydroxyl groups, facilitating better incorporation into the hydrogel network and more efficient crosslink formation.^[^ ^9^, ^13^, ^33^ ^]^ Elongation at break (Figure S3, Supporting Information) was highest for OL hydrogels (O‐5%: 41.95 ± 0.002 mm), further highlighting the material balance of strength and flexibility.

KL higher swelling tendency (Figure 1d) likely contributes to reduced mechanical integrity, especially at lower crosslinker levels, due to network loosening from water absorption. These findings suggest that optimization of crosslinking and lignin selection can yield mechanically robust hydrogels suitable for tissue engineering applications. While the mechanical properties of the current lignin‐based hydrogel do not match those of high‐load‐bearing tissues, this work introduces a novel, scalable, and sustainable route to fabricate a tubular hydrogel from lignin. These findings lay the groundwork for further optimization and application‐specific modification, particularly in areas such as soft tissue engineering, drug delivery, and bio‐scaffold development.

The 100‐cycle tensile fatigue analysis revealed that the fatigue resistance of lignin hydrogels is highly dependent on crosslinking density (Figure S4, Supporting Information). Hydrogels with low (2.5%) and high (10%) crosslinker content exhibited reduced mechanical performance, likely due to insufficient network formation in the former and excessive rigidity with internal stress buildup in the latter. In contrast, the K‐5% and O‐5% samples demonstrated superior fatigue resistance, maintaining consistent stress levels and Young's modulus across cycles, with minimal hysteresis and structural degradation (Table S1, Supporting Information). This near‐elastic response suggests effective energy dissipation and network stability under repetitive strain. The results underscore the importance of optimized crosslinking to balance flexibility and mechanical robustness, a critical requirement for load‐bearing biomedical applications such as tendon scaffolds.

Furthermore, hydrogels with lower crosslinking showed greater swelling, suggesting higher degradation potential due to looser network structures. To evaluate mechanical durability during potential network relaxation, K‐5% and O‐5% hydrogels were subjected to 1,000‐cycle tensile fatigue testing (Figure S5, Table S2, Supporting Information). Both samples maintained structural integrity throughout the test. K‐5% showed a relatively stable Young's modulus across cycles, while O‐5% exhibited an initial decrease followed by partial recovery, indicating differences in network adaptation under cyclic loading. These findings suggest that both hydrogels demonstrate promising short‐term mechanical resilience, supporting their potential in load‐bearing applications. Further research involving long‐term degradation studies under physiological conditions is needed to establish a direct correlation between structural breakdown and mechanical performance.

Strain energy function (SEF) modeling using the Yeoh model was applied to characterize the nonlinear elastic behavior of lignin–PVA hydrogels under uniaxial tensile loading.^[^ ^34^, ^35^ ^]^ Based solely on the first strain invariant (*I*_1_), the Yeoh model provided an excellent fit across all formulations and crosslinker concentrations, capturing the hydrogels’ mechanical response with high fidelity (Figure 2f). Its three‐parameter formulation achieved low percentage errors (1.03–3.04%) and strong cumulative R^2^ values, indicating robust predictive capability. Model accuracy improved with increasing crosslinking, consistent with enhanced stiffness and network uniformity; for example, the O‐10% hydrogel exhibited the best agreement with experimental data, showing only 1.03% error. These findings confirm the Yeoh model's effectiveness for representing the mechanical performance of lignin‐reinforced hydrogels within physiologically relevant strain ranges. This predictive modeling approach offers a valuable tool for integrating these materials into computational design and simulation pipelines for soft tissue engineering applications.

**Figure 3:**
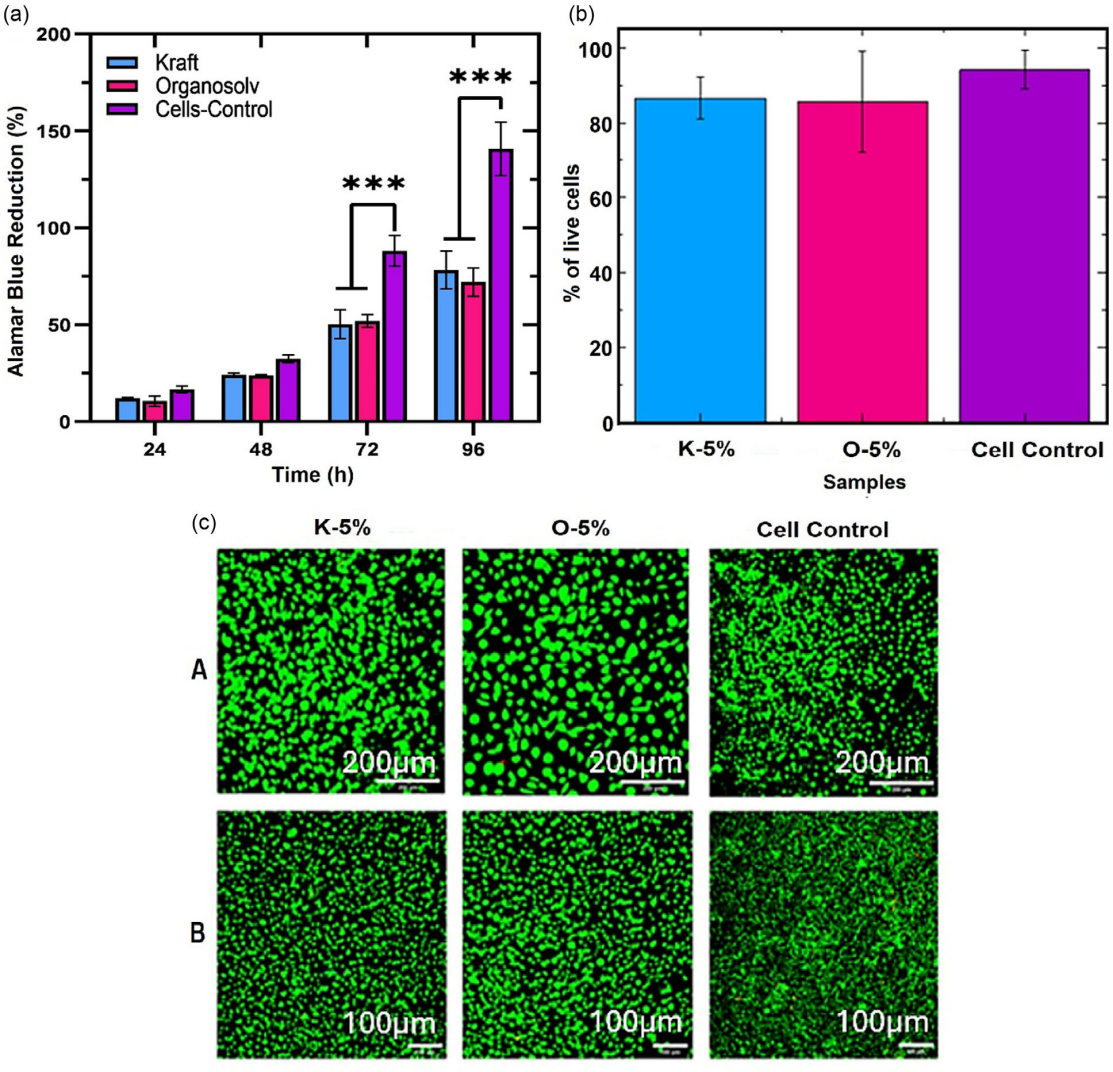
a) Cytotoxicity of fibroblasts measured by means of Alamar Blue Proliferation Assay cultured 96 h in the presence of kraft and lignin hydrogels at 5% ECH concentration, b) quantification of cellular viability from LIVE/DEAD assay, gathered by means of ImageJ from c) LIVE/DEAD staining of fibroblasts cultured in lignin hydrogels media for 96 h, A) scale bar‐200 μm, B) scale bar‐100 μm.

The cytocompatibility of lignin‐based hydrogels intended for soft tissue regeneration was assessed by employing the Alamar Blue (AB) assay to quantify fibroblast proliferation over a 96‐hour incubation period (**Figure** **3**a). L929 fibroblasts, a standard cell line for cytotoxicity screening, were exposed to media conditioned with K‐5% and O‐5% hydrogels.^[^ ^36^ ^]^ While both hydrogel groups demonstrated a sustained increase in metabolic activity over time, the proliferation rates remained statistically lower than the cell‐only control, particularly at 72 and 96 h (p < 0.01). For instance, at 72 h, the K‐5% and O‐5% groups exhibited AB reductions of 50.2 ± 7.4% and 51.8 ± 3.3%, respectively, compared to 88.2 ± 7.6% for the control. This attenuated proliferation is likely a result of lignin's inherently low cell adhesion profile due to its hydrophilicity and absence of cell‐binding moieties.^[^ ^36^ ^]^ Nevertheless, no cytotoxic effects were observed, indicating that the materials are cytocompatible and capable of maintaining cell viability. Two‐way ANOVA showed a statistical difference (*** p ≤ 0.001) in the proliferation rates of cells between the two lignin hydrogel groups and the cell control at time points 72 and 96 h, though no statistical difference was found between the lignin hydrogel groups themselves.

Further investigation for the metabolic assay data and the cell‐material interactions, a LIVE/DEAD fluorescence assay was conducted after 96 h of cell culture (Figure 3b). Confocal microscopy revealed predominantly viable fibroblasts, evidenced by widespread green fluorescence with negligible red staining, confirming the absence of acute cytotoxicity (Figure 3c). Quantitative viability analysis via ImageJ revealed high survival rates for both K‐5% (86.5 ± 5.6%) and O‐5% (85.5 ± 13.5%), closely mirroring the control group (94.1 ± 5.1%). Morphologically, fibroblasts displayed a typical spread with elongated extensions and spindle‐shaped bodies, an indicator of active cytoskeletal organization and anchorage‐dependent behavior.

These results suggest that both Kraft and Organosolv lignin‐based hydrogels possess baseline biocompatibility suitable for use in soft tissue engineering scaffolds. The similar performance of both lignin types implies that lignin origin does not substantially influence short‐term cytocompatibility, although further biological investigations will need to be conducted to assess the biological response more fully. Moreover, the hydrogel's ability to support cell viability and maintain phenotypic morphology under static conditions provides a promising foundation for further optimization. Enhancing cell adhesion through surface functionalization, incorporation of bioactive peptides, or ECM‐mimetic coatings may further improve cellular integration and regenerative potential. Such modifications are particularly relevant for load‐bearing or mechanically dynamic environments, where scaffolds must balance structural performance with biological responsiveness.

**Figure 4:**
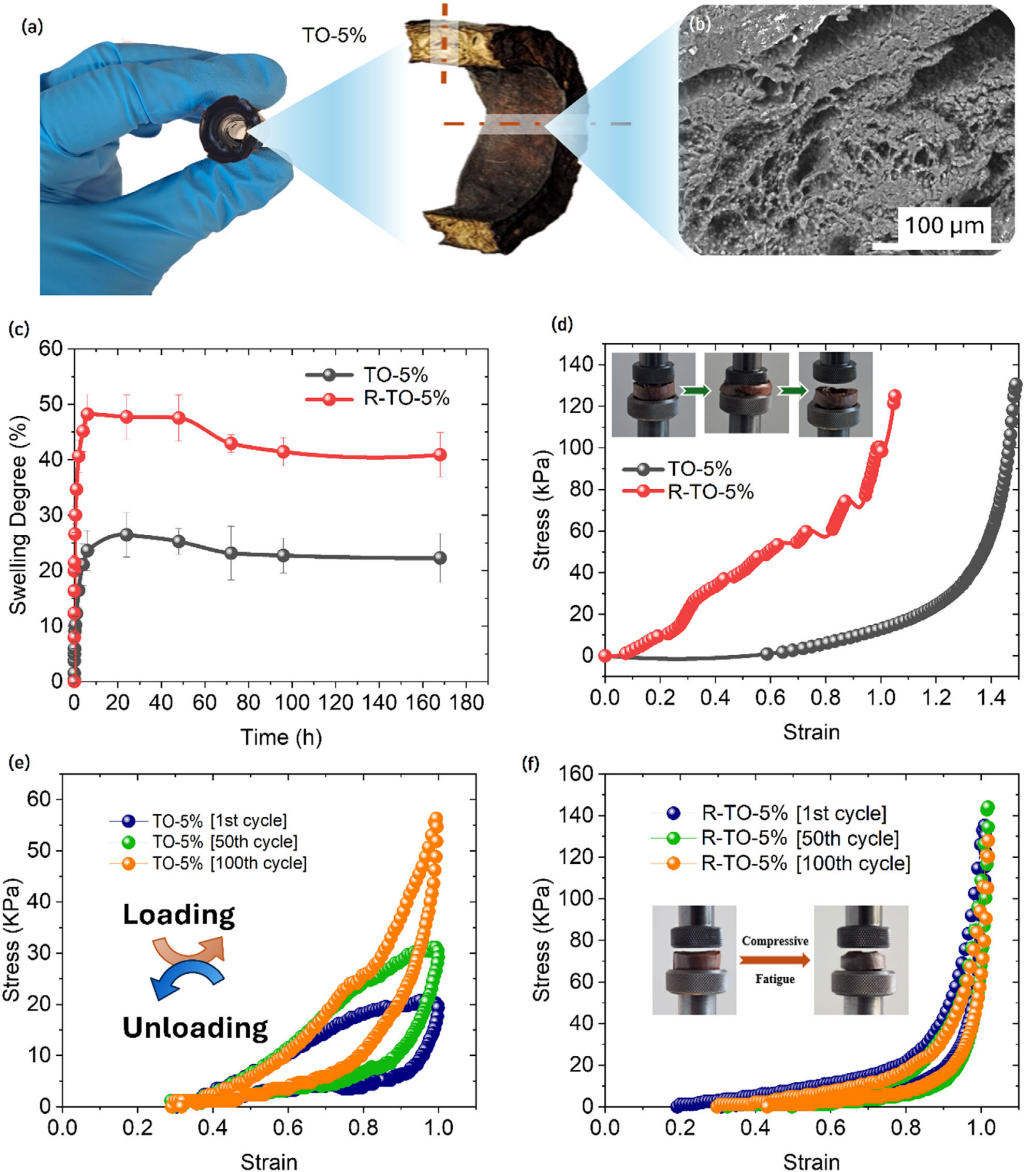
Lignin‐based tubular hydrogel. a) Digital image and cross‐sectional view of the freeze‐dried sample (TO‐5%), b) morphological characterization using SEM, c) comparison of tubular hydrogels with and without reinforcement in terms of swelling capacity, d) compressive stress, and e,f) cyclic compression fatigue performance.

Based on the comprehensive analysis of chemical, mechanical, swelling properties, and biocompatibility, the O‐5% hydrogel was selected for fabrication into tubular scaffolds, both with (R‐TO‐5%) and without (TO‐5%) reinforcement of polypropylene (PP) mesh, to assess its performance in configurations relevant to tendon tissue engineering applications (**Figure** **4**a). SEM analysis, as shown in Figure 4b and Figure S6, Supporting Information, characterized the surface and internal morphologies of the TO‐5% and R‐TO‐5% hydrogels. Both hydrogels exhibited isotropic porous architectures with uniformly distributed pores and no preferential orientation throughout the cross‐sections. PP filaments in the R‐TO‐5% samples appeared as smooth, nonporous features. Limited interfacial bonding between the hydrophilic hydrogel matrix and hydrophobic PP mesh resulted in pronounced voids surrounding the filaments. Porosity was notably reduced near the sample boundaries interfacing with the mold, generating densified regions at the inner and outer edges. Similar densification was observed adjacent to the PP filaments, indicating localized structural compaction. The larger pores surrounding the mesh in R‐TO‐5% constructs could potentially facilitate localized accelerated degradation, allowing earlier exposure of reinforcing filaments to soft load‐bearing tissue and thereby enhancing mechanical stability during tissue repair. Additionally, the smooth, pore‐free external surfaces observed in digital images may reduce undesired interactions with surrounding tissues, contributing to scaffold durability.

The swelling behavior of both hydrogels followed a similar kinetic profile, reaching equilibrium within 24 h of immersion in PBS (Figure 4c). At saturation, the TO‐5% samples exhibited a swelling degree of 26.43 ± 5.64%, while the R‐TO‐5% samples showed a higher value of 32.27 ± 3.94%. This enhancement is attributed to the embedded mesh, which introduced larger interconnected pores within the hydrogel network, promoting faster fluid diffusion throughout the matrix. The R‐TO‐5% samples demonstrated a steeper initial swelling rate and greater equilibrium hydration. Over the 168‐hour observation period, a gradual reduction in swelling degree was noted, with R‐TO‐5% constructs showing slightly faster decline, likely due to mesh‐induced localized porosity. Notably, these reinforced samples retained a relatively high residual swelling degree (25.37%) at 168 h, indicating sustained fluid retention and structural stability. Further long‐term and in vivo studies are necessary to confirm degradation dynamics and scaffold performance under physiological conditions.

Mechanical testing under compression (Figure 4d) showed marked differences between TO‐5% and R‐TO‐5% hydrogel. The TO‐5% samples with tubular morphology and a central cavity exhibited reduced stiffness and compressive strength, highlighting the influence of hollow geometry on mechanical performance. In contrast, the R‐TO‐5% hydrogels, reinforced with a PP mesh, displayed a biphasic stress–strain profile with two distinct compressive moduli of 135.08 ± 36.45 and 351.98 ± 150.06 kPa. The initial modulus exceeded that of the TO‐5% control by roughly 10 times, indicating effective mechanical reinforcement. The slope transition suggests decoupling between the hydrogel and mesh under strain, with the latter bearing increasing load in the second phase. Importantly, R‐TO‐5% constructs retained structural integrity after full compression, showing only minor mesh deformation. These results confirm that mesh reinforcement enhances both stiffness and resilience, supporting the design suitability for load‐bearing tissue scaffolds.

Cyclic compression tests (Figure 4e,f) were performed over 100 cycles to evaluate the mechanical durability and fatigue resistance of the tubular hydrogels with and without reinforcement. The TO‐5% samples exhibited a significant increase in maximum stress over repeated cycles, with the 100th cycle reaching ≈60 kPa at 100% strain, indicating strain hardening due to structural compaction and densification of the porous matrix. However, these samples also showed considerable hysteresis between loading and unloading curves, suggesting pronounced energy dissipation and plastic deformation under cyclic loading.^[^ ^37^ ^]^ In contrast, the R‐TO‐5% hydrogels demonstrated a markedly higher initial compressive strength (≈140 kPa at 100% strain in the 100th cycle) and maintained consistent stress–strain profiles across repeated cycles, indicative of superior fatigue resistance and structural resilience. The embedded PP mesh in R‐TO‐5% likely restricted permanent deformation and facilitated elastic recovery by distributing the compressive load more uniformly. Minimal change in curve shape across cycles suggests that the reinforcement effectively preserved mechanical integrity during long‐term mechanical stress, making R‐TO‐5% hydrogels promising candidates for load‐bearing tissue engineering applications such as tendon scaffolds.

## 3 Conclusions

This study demonstrates the feasibility of developing lignin–PVA hydrogel scaffolds with tailored mechanical, chemical, and biological properties for biomedical applications. Organosolv lignin, particularly at 5% ECH crosslinking, achieved optimal performance with a UTS of 83.14 ± 0.16 kPa and Young's modulus of 39 ± 8 kPa. Tubular hydrogel scaffolds reinforced with PP mesh (R‐TO‐5%) displayed a biphasic compressive profile with moduli of 135.08 ± 36.45 and 351.98 ± 150.06 kPa and maintained structural integrity over 100 loading cycles, indicating excellent fatigue resistance. Cytocompatibility was confirmed by cell viability rates above 85% after 96 h. These results highlight the potential of lignin‐based scaffolds as sustainable and modular platforms for applications such as tendon repair, vascular grafts, and nerve regeneration. While promising performance was observed under static and cyclic loading in the dry state, future work will evaluate swollen‐state mechanics to better reflect physiological conditions. Further in vivo validation is necessary to assess biodegradation, immune response, and long‐term integration. Surface functionalization with ECM proteins or bioactive peptides may enhance cell adhesion, while dynamic bioreactor or coculture models could better simulate physiological conditions and guide scaffold optimization. These future directions will help transition lignin‐based scaffolds from proof‐of‐concept to clinically viable solutions.

## 4 Experimental Section

4.1

4.1.1

##### Materials

Alcell organosolv (O, Mw 4000 g/mol) and Kraft lignin (K, Mw 3153 g/mol) were sourced from Tecnaro GmbH (Germany). Sodium hydroxide (≥98%), PVA(99% hydrolyzed, Mw 85 000–124 000 g/mol), and ECH ≥99% were purchased from Sigma‐Aldrich (USA). PBS was obtained from Sigma‐Aldrich (UK), and PP mesh for reinforcement from Easy Composites EU (Netherlands). For cell culture, DMEM, L‐Glutamine, Penicillin‐Streptomycin, propidium iodide, and L929 fibroblasts were from Sigma‐Aldrich (Ireland), while Alamar Blue and Calcein AM were sourced from Invitrogen (ThermoFisher, Ireland).

##### Hydrogel Synthesis

Hydrogels were prepared using both types of lignin with varying concentrations of ECH as a crosslinker (2.5–10%v/v). A 16% PVA solution and 2 M NaOH were prepared in deionized water. Equal volumes of PVA and NaOH were mixed, followed by the addition of lignin (0.9 g per 10 mL). After homogenization, ECH was added dropwise at a rate of one drop per second, and the mixture was stirred at 750 rpm for 30 min before being cast into molds. The samples were then left to crosslink overnight at room temperature., then washed with acetone and deionized water, and finally dried. Hydrogels were labeled as K‐y% or O‐y%, based on lignin type and ECH concentration. For tubular hydrogels, both reinforced and non‐reinforced samples were synthesized by pouring the hydrogel solution into cylindrical molds containing a central shaft to form a hollow core. For reinforced samples, PP mesh was positioned in the mold prior to casting.

##### Materials Characterizations

The morphology of the lignin–PVA hydrogels, scanning electron microscopy was performed using a Hitachi SU‐70 Field Emission SEM (Bernal Institute, University of Limerick, Ireland). Samples were frozen for 24 h, followed by freeze‐drying for an additional 24 h using a Martin Christ Alpha 2–4 LSCplus freeze dryer to ensure complete dehydration. Dried samples were mounted on 15 mm aluminum stubs using carbon tape, then sputter‐coated with gold for 30 s using an Emi‐Tech K550 sputter coater to improve conductivity. Imaging was conducted at an accelerating voltage of 10 kV and a working distance of 10 mm. FTIR was used to analyze the chemical composition of the hydrogels and their raw components, using a PerkinElmer FTIR spectrometer (Bernal Institute, University of Limerick) over a spectral range of 4000–650 cm^−1^. Prior to FTIR analysis, samples were oven‐dried at 60 °C to remove moisture. Swelling capacity was assessed by immersing preweighed, oven‐dried hydrogel discs in PBS at 37 °C, with weights measured at defined intervals up to four months using an analytical balance (five‐decimal precision). Compression testing was carried out using a Zwick/Roell Z010 universal testing machine equipped with a 200 N load cell. Hydrogel discs were compressed at 10 mm min^−1^ to 40% strain to determine compressive modulus and stress–strain behavior. Tensile properties were evaluated using dogbone‐shaped hydrogel specimens under the same machine setup, pulled at 10 mm/min until failure, with thickness and gauge length measured using digital calipers.

For cell culture, lignin hydrogels were sterilized UV sterilization and immersed in DMEM cell culture media in order to reach equilibrium for 72 h at 37 °C. L929 fibroblasts were grown in DMEM supplemented with 10% fetal bovine serum, 1% L‐Glutamine, and 1% Penicillin‐Streptomycin in a 5% CO_2_ environment.

For all biological tests, the L929 fibroblast (P8) cell cultures were seeded at a density of 0.05 × 106 cells/well in a 24‐well plates and allowed to attach overnight, followed by supplementation with 1 mL of lignin hydrogel‐treated cell culture media.

LIVE/DEAD staining of the cells was conducted after 96 h by means of Calcein AM and propidium iodine and imaged using an ImageXpress confocal microscope for fluorescence imaging (Molecular Devices, USA). The cell viability was then quantitatively analyzed using ImageJ.

Moreover, ligning hydrogel samples were tested for potential cytotoxicity by means of the Alamar Blue proliferation assay. Alamar Blue was added to the wells at 10% of the well volume and incubated for 5 h. Measurement of the cell fluorescent emission was carried out using SynergyMx (BioTek, UK) at a wavelength of 545/590 nm, following the manufacturer's protocol.

For the cellular analysis, experiments were conducted in triplicates, with the data presented as mean ± standard deviation. To determine the statistical significance, two‐way ANOVA was employed for the Alamar Blue cytocompatibility analysis, with *p*‐values denoted as follows: * p ≤ 0.05, ** p ≤ 0.01, and *** p ≤ 0.001.

##### Strain Energy Function

In this study, the Yeoh model was selected for its proven effectiveness in modeling the mechanical behavior of soft materials such as reinforced rubbers, biological tissues, and hydrogels. The Yeoh model expresses the strain energy density function (*W*) in terms of the first strain invariant (*I*_1_).

(1) $$W=C_{10}\left(I_{1}-3\right)+C_{20}{\left(I_{1}-3\right)}^{2}+C_{30}{\left(I_{1}-3\right)}^{3}$$

where *C*_10_, *C*_20_, and *C*_30_ are material constants determined through experimental data fitting. For uniaxial tension, assuming incompressibility, the principal stretches are taken as $\lambda_{1}=\lambda$, $\lambda_{2}=\lambda_{3}=\lambda^{-1/2}$, and the first invariant becomes.

(2) $$I_{1}=\lambda^{2}+2\lambda^{-1}$$

Substituting into the Yeoh model and differentiating with respect to *λ* yields the nominal stress.

(3) $$\sigma_{e}=(2\lambda -2/\lambda^{2})\times \left[C_{10}+2C_{20}\left(\lambda^{2}+2/\lambda -3\right)+3C_{30}{\left(\lambda^{2}+2/\lambda -3\right)}^{2}\right]$$

This expression was used to evaluate the stress‐stretch behavior of hydrogel samples. The material constants were obtained by fitting the model to experimental data using Excel Solver. The fit quality was assessed using a regression coefficient

(4) Regression (R)=Model stress/Average Experimental

and the percentage error calculated by.

(5) $$\%\text{Error= }\left(\text{Average}\;\text{of}\;\text{Sum}\;\text{of}\;R^{2}\right)\times 100$$

Based on regression accuracy and physical realism, the Yeoh model provided the most consistent and accurate fit across the tested deformation range. Comprehensive details for materials and modeling can be found in supplementary section.

## Conflict of Interest

The authors declare no conflict of interest.

## Supporting information

Supplementary Material

## Data Availability

The data that support the findings of this study are available from the corresponding author upon reasonable request.
