# Supplementary material for: Engineering Lignin‐Based Tubular Hydrogel Scaffolds for Load‐Bearing Biomedical Applications
Source: ChemSusChem. 2025 Sep 11;18(20):e202501520. doi: 10.1002/cssc.202501520 (PMC12548947; doi:10.1002/cssc.202501520)
Supplement: Supplementary file 1 — Supplementary Material [file CSSC-18-e202501520-s001.pdf]

eSupporting Information

Engineering Lignin-Based Tubular Hydrogel Scaffolds for Load-Bearing Biomedical Applications

Muhammad Muddasar[a,*], *Grace Joyce*[a], *Mathilde Pouzier*[a], *Aleksandra Serafin*[a], *and Maurice N. Collins*[a,c,*]

[a] Stokes Laboratories, School of Engineering, Bernal Institute, University of Limerick, Limerick, Ireland

[b] Advanced Materials and Bioengineering Research (AMBER) Centre, Ireland

*Corresponding author.

E-mail address: Muhammad.muddasar@ul.ie (M Muddasar); Maurice.collins@ul.ie (M.N. Collins)

Material Characterisation

1.1.1. Scanning Electron Microscopy

Scanning Electron Microscopy (SEM) is a widely used technique for analysing surface morphology of samples with high resolutions and magnifications. It works by directing a focused beam of high-energy electrons onto a material's surface, producing signals such as secondary electrons, backscattered electrons, and X-rays that provide information about topography, composition, and other structural features. Compared to optical microscopy, SEM offers significantly higher resolution, typically ranging from < 1 nanometre to several nanometres. The magnification in an SEM can be adjusted across approximately six orders of magnitude, ranging from around 10x to 3,000,000x. An SEM system consist of the following components; an electron gun which is the source of high-energy electrons, condenser and objective lenses which control and focus the electrons, scan coils and generator that direct the electron beam across the sample surface, detectors that capture signals produced by electron-sample interactions, sample stage that holds and positions the specimen within the vacuum system which maintains necessary conditions for electron beam stability, and a computer with a display to process the detected signals and generate grey-scale real-time images for analysis [1, 2].

The SEM instrument (Hitachi SU-70, Bernal Institute, University of Limerick, Ireland) was used to examine the morphology of the 3D printed Lignin-PVA samples. To prepare the samples for analysis, samples were placed in the freezer for 24 hrs and then freeze-dried for 24 hrs (Martin Christ Alpha 2-4 LSCplus, Bernal Institute, University of Limerick, Ireland). This was important to get the required dryness for entry into the SEM, since the apparatus used a vacuum system. The samples were mounted to carbon tape on a 15 mm aluminium stub. Following that, they were sputter-coated with gold for 30 seconds using the Emi-Tech K550 sputter coater for optimal conductivity. Subsequently, they were analysed using the SEM. A 10 kV accelerator voltage was used to assess the dispersion of the extruded material on the printed samples at a working distance of 10 mm. This environment facilitated ideal imaging settings for precise analysis.

1.1.2. Fourier Transform Infrared Spectroscopy

The physicochemical characteristics of lignin-PVA-based hydrogels and their raw materials were analysed using Fourier Transform Infrared (FTIR) spectroscopy (Bernal Institute,

University of Limerick) in a PerkinElmer. For the analysis of lignin-PVA hydrogels, FTIR provides a non-destructive method to examine the key structural features of each component and their interactions within the hydrogel matrix. This technique offers several advantages, including high sensitivity and selectivity, and efficient data processing. [3]. For each test, four scans in the range of 4000–650 cm^{-1} were performed. Prior to analysis, the hydrogels were oven-dried at 60 °C to remove excess moisture.

Transmittance is the fraction of incident infrared radiation that passes through a sample without being absorbed. Transmittance values range from 0% (no transmission, complete absorbance) to 100% (complete transmission, no absorbance) [4].

1.1.3. Mechanical Testing

1.1.3.1. Swelling Capacity

The water absorption test was conducted to determine the water uptake by the samples over time under specific conditions. This test also provided insights into the porosity of the samples. A total of eighteen discs (triplicates of each sample) were used. Initially, the dry weight (Wd) of each disc was measured using an analytical balance with five-decimal precision. The wet weight (Ws) of each sample was later compared to its original dry weight, which served as the baseline.

To ensure thorough drying before testing, the samples were placed in an oven at 50 °C until a stable weight was recorded. Each sample was then submerged in phosphate-buffered saline (PBS) within a sealed plastic container. To maintain consistency, the PBS-filled containers were preheated in a water bath at 37 °C (FisherbrandIsotemp GPD 05, University of Limerick, Ireland), with a thermometer used to verify temperature accuracy, for 24 hrs before testing. The sealed containers prevented water evaporation or contamination.

The weight of the water-absorbed samples was measured at multiple time points: 20 min, 40 min, 1 hr, 2 hrs, 3 hrs, 4 hrs, 1 day, 2 days, 3 days, 1 wk, 2 wk, 3 wk, 1 mth, 2 mths, 2 mths, 3 mths, and 4 mths. At each interval, the samples were removed from the water, gently dried with blotting paper to remove surface moisture, and immediately weighed to determine their wet weight. All measurements were recorded in triplicate for accuracy. To ensure consistency in subsequent measurements, the exact time of sample removal and re-immersion in the water bath was documented.

The % swelling of the samples was then calculated using Equation 1:

$$\% \, swelling = \frac{(Ws - Wd)}{Wd} \, x \, 100 \qquad \textit{Equation 1}$$

Where Wd is the weight of the dry hydrogel (g) and Ws is the weight of the swollen hydrogel (g) at each time interval.

1.1.3.2. Compression Testing

Mechanical properties were assessed through tensile, compressive, fatigue, and creep tests, all conducted using a Zwick/Roell Z010 10KN Proline equipped with a 200 N load cell. The machine was set to record force (N), displacement (mm), time (s), cycle number, outputting data for further analysis. All tests were performed at a constant temperature with a preload of 0.005 N applied before testing.

A total of 18 hydrogel disc samples (triplicates of each type) were tested. Each sample was placed between two plates and compressed at a standard compression rate of 10 mm/min to 40% strain. Cylindrical specimens (diameter = 10 mm, height = 10 mm) were tested at a strain rate of 10 mm/min. This strain was selected based on previous research, which indicates that tendon failure typically occurs at strains above 8-10%, as well as the limitations imposed by the sample thickness in the compression test apparatus[5]. Key mechanical properties analysed included the yield point, elastic limit, stress-strain relationship, Young's modulus €, and compressive strength. The force and displacement data obtained allowed for the generation of stress-strain curves, from which E was determined.

Equation 2 is used to calculate Engineering Stress (σ_e):

$$\sigma_e = \frac{F}{A_o} \qquad \textit{Equation 2}$$

Where $A_o$is the sample's original cross-sectional area (in this example, the area of a circle) and F is the applied force:

$$A_o = \pi r^2 \qquad \textit{Equation 3}$$

Where r is the sample's radius.

Equation 4 is used to calculate Strain (ε):

$$\varepsilon = \frac{\Delta L}{L} \qquad \textit{Equation 4}$$

Where L is the original sample length (in this example, its height) and ΔL is change in displacement.

Using the answers calculated from Equation 2 and 4, the Young's Modulus (E) can be obtained:

$$E = \frac{\sigma_e}{\varepsilon}$$ *Equation 5*

E was obtained from the linear region of the stress-strain curve, selecting the range where the regression value was closest to 1. Standard deviation was calculated, and error bars were included in the bar charts to indicate variability between samples.

1.1.3.3. Tensile Testing

Tensile tests were conducted on dumbbell-shaped lignin–PVA hydrogel samples cast according to ISO 23529:2016, with a gauge length of 9.53 mm and a thickness of approximately 3 mm. Prior to testing, sample thickness and gauge length were measured using a vernier caliper. Each sample was clamped proximally and distally at the ends of the dumbbell geometry, and a preload of 0.005 N was applied to eliminate slack. Tensile testing was performed at a crosshead speed of 10 mm/min at ambient temperature (~23 ± 2 °C) using a Zwick/Roell Z010 ProLine universal testing machine equipped with a 200 N load cell. The mechanical properties were assessed by analyzing the stress–strain response, and all measurements were conducted in triplicate to ensure reproducibility.

E values were determined using the method described in section 1.1.3.2, Where A_o is the sample's original cross-sectional area (in this example, the area of a rectangle).

$$A_0 = Gauge\ Length\ Depth\ x\ Gauge\ Length\ Width$$ *Equation 6*

Strain Energy Function

Material characterisation plays a fundamental role in biomedical engineering, particularly in the design and optimisation of biomaterials such as hydrogels for medical applications. Hydrogels exhibit viscoelastic behaviour, possessing both solid-like and fluid-like characteristics. To predict their mechanical response under different loading conditions, strain energy functions (SEFs) are used. These functions mathematically describe how materials store and dissipate strain energy upon deformation.

Hyperelastic materials can stretch beyond 50% of its original length under load, a behaviour that can only be accurately modelled using nonlinear methods that account for finite deformations rather than the infinitesimal deformations assumed by linear mathematical approaches. As a result, Hooke's law is insufficient for describing the mechanical behaviour of hydrogels [6]. A hyperelastic material is characterised by a nonlinear constitutive SEF

equation, also referred to as a stored energy density function. SEFs relate strain energy to the principal stretches derived from mechanical testing.

Since hydrogels can experience various deformation modes, three primary strain types of tension, shear, and compression, are typically required to fully characterise their mechanical response. However, in certain cases, not all three strain types can be experimentally measured, necessitating the selection of an appropriate SEF that best fits the available data.

Several SEFs have been developed for modelling hyperelastic materials, each tailored to specific loading conditions. The most commonly used SEFs include:

1. Neo-Hookean
2. Mooney-Rivlin
3. Signorini Form
4. Yeoh Form
5. Delfino Type
6. Fung Type
7. Vaishnav Type (2D)
8. Hayashi Type (2D)

The selection of an SEF is based on its ability to accurately replicate experimental stress-strain behaviour. Each model has unique characteristics that influence its suitability for a given material and testing scenario.

The material constants $C_{10}, C_{20},$ and C_{30} were found using the data analysis solver on Excel and used to solve Equation 7and 8.The performance of different SEFs was assessed by comparing their regression fits and percentage errors relative to experimental data.

$$Regression\ (R) = \frac{Model - Average\ Stress}{Average\ Stress}$$ *Equation 7*

$$\%\ Error = (Average\ of\ Sum\ of\ R^2)\ x\ 100$$ *Equation 8*

Derivations

For materials exhibiting significant elongation, the stretch ratio (λ) is the most suitable parameter. The stretch ratio is defined as the ratio of the final length (L_f) to the original length (L_o) of the sample.

$$\lambda = \frac{L_f}{L_o}$$ *Equation 9*

The zwich measures displacement and force, which may be used to determine the stretch ratio. By using the equations outlined in section 1.1.3.2. we can define the relationship is as follows:

$$\lambda = 1 + \varepsilon$$ *Equation 10*

The Yeoh model has been used to characterise the behaviour of many hyperelastic materials across literature, including reinforced natural rubber, hydrogels, and biological tissues [7, 8]. The Yeoh Model is derived as follows:

$$W = C_{10}(I_1 - 3) \quad + C_{20}(I_1 - 3)^2 + C_{30}(I_1 - 3)^3$$ *Equation 11*

Where I_1 and I_2 are the first and second strain invariants respectively of the Right-Cauchy strain tensor:

$$I_1 = {\lambda_1}^2 + {\lambda_2}^2 + {\lambda_3}^2$$ *Equation 12*

$$I_2 = {\lambda_1}^2{\lambda_2}^2 + {\lambda_2}^2{\lambda_3}^2 + {\lambda_3}^2{\lambda_1}^2$$ *Equation 13*

Where $\lambda_1$is the principal stretch ratio and C_{10}, C_{20}, and$C_{30}$are material constants.

Where:

$$1 = \lambda_1 + \lambda_2 + \lambda_3$$ *Equation 14*

$$\lambda_1 = \lambda$$ *Equation 14. 1*

$$\lambda_2 = \lambda_3$$ *Equation 14. 2*

Putting the values in Eq. 12 and 13:

$$\therefore I_1 = \lambda^2 + \frac{2}{\lambda}$$ *Equation 12. 1*

$$\therefore I_2 = 2\lambda + \frac{1}{\lambda^2}$$ *Equation 13. 1*

With the following correlations Equation 11 can be rewritten as,

$$W = C_{10}\left(\lambda^2 + \frac{2}{\lambda} - 3\right) + C_{20}\left(\lambda^2 + \frac{2}{\lambda} - 3\right)^2 + C_{30}\left(\lambda^2 + \frac{2}{\lambda} - 3\right)^3$$ *Equation 11. 1*

The Yeoh model is then differentiated in respect to the λ to give σ_e and then simplified:

$$\sigma_e = \frac{dW}{d\lambda} = C_{10}\left(2\lambda - \frac{2}{\lambda^2}\right) + 2C_{20}\left(\lambda^2 + \frac{2}{\lambda} - 3\right)\left(2\lambda - \frac{2}{\lambda^2}\right) + 3C_{30}\left(\lambda^2 + \frac{2}{\lambda} - 3\right)^2\left(2\lambda - \frac{2}{\lambda^2}\right)$$ *Equation 11. 2*

$$\frac{dW}{d\lambda} = \left(2\lambda + \frac{2}{\lambda^2}\right)\left(C_{10} + 2C_{20}\left(\lambda^2 + \frac{2}{\lambda} - 3\right) + 3C_{30}\left(\lambda^2 + \frac{2}{\lambda} - 3\right)^2\right)$$ *Equation 11. 3*

1.1.4. In-vitro biocompatibility of hydrogels

Viability assays yield relative or absolute assessments of cellular proliferation or cytotoxicity, rendering them suitable for evaluating the safety and efficacy of implantable materials. Direct viability measures offer absolute quantifications through selective stains or markers that differentiate live from dead cells. Indirect viability assays depend on reporter molecules, whose intensity can be contrasted with a control for relative variations in cell viability or against a calibration curve to estimate the number of viable cells [9].

Cellular metabolic activity is a key indicator for assessing cytotoxicity, viability, and proliferation within a cell population. Metabolically active cells maintain a reducing environment within their cytosol, which can be leveraged using colourimetric or fluorometric redox indicators. These indicators undergo a measurable conversion, allowing for spectrophotometric analysis of metabolic function. The Alamar Blue™ (AB) assay is a widely used metabolism-based technique that employs resazurin (7-hydroxy-3H-phenoxazin-3-one 10-oxide), a blue, non-fluorescent molecule. Once internalized by viable cells, resazurin is reduced to resorufin (7-hydroxy-3H-phenoxazin-3-one), a highly fluorescent red compound, due to the intracellular reducing conditions. This transformation is mediated by intracellular diaphorases, which utilise Nicotinamide Adenine Dinucleotide Phosphate (NADPH) and Nicotinamide Adenine Dinucleotide (NADH) as reducers. Resorufin exhibits strong fluorescence, with an excitation range of 530–570 nm and an emission range of 580–610 nm, making it a reliable indicator of cell viability. Additionally, the assay can be analysed via absorbance at 570 nm, using 600 nm as a reference wavelength to normalise data [10].

Resazurin and resorufin are water-soluble, stable in culture media, non-toxic, and membrane-permeable, making the AB assay a robust, cost-effective, and easy-to-perform alternative to other cell viability and proliferation tests. This assay has been extensively used to evaluate cytotoxicity across mammalian cells, cell lines, bacteria, and fungi, demonstrating its reliability across various biological systems Despite its advantages, several factors must be considered when interpreting AB assay results. First, the assay does not directly count cells, meaning its fluorescence or absorbance signal reflects both viable cell quantity and metabolic activity. As a result, the data represents the metabolic state of the entire population rather than individual cells. Impaired or non-viable cells exhibit reduced metabolism, leading to a weaker

resorufin signal. However, changes in cell proliferation do not always indicate cell death, as certain test compounds may enhance or inhibit proliferation rather than induce cytotoxicity. Thus, careful interpretation is required, as the AB assay primarily reflects overall metabolic activity, which may be influenced by cell viability, proliferation, or both. To accurately assess cytotoxicity, the AB assay is usually complemented with additional tests such as a LIVE/DEAD assay [10].

Calcein-AM staining is a widely used method for assessing cell viability in a LIVE/DEAD assay, relying on intracellular esterase activity to convert Calcein-AM into its fluorescent form, Calcein. Calcein-AM (Calceinacetomethoxy) is a non-fluorescent, membrane-permeable dye commonly used in cytotoxicity assays. Once inside viable cells, it undergoes hydrolysis by cytoplasmic esterases, producing Calcein, a negatively charged, membrane-impermeable molecule that remains trapped within the cytoplasm. The accumulation of Calcein generates a strong green, fluorescent signal, directly proportional to the number of viable cells. In contrast, nonviable cells lack esterase activity, preventing dye retention and resulting in an absence of fluorescence. To simultaneously assess cell death, Calcein-AM is often paired with Propidium Iodide (PI), a membrane-impermeable dye that penetrates only compromised, nonviable cells, producing a red fluorescent signal upon binding to DNA. This dual-staining approach enables quantification of both live and dead cells, making it a highly effective technique for evaluating cell viability and cytotoxicity in culture systems [11].

For cell culture studies, the protocol published by Serafin *et al.,* [12] was closely followed using KL and OL hydrogels at the optimal crosslinker concentration based off mechanical testing results. Materials were sterilised using autoclaving, followed by UV sterilisation for 30 min, and submerged in Dulbecco's Modified Eagle Medium (DMEM) cell culture media to achieve equilibrium for 72 hours at 37 °C. Due to their high-water content, hydrogel-based samples required both autoclaving and UV sterilisation. However, no specific antimicrobial testing was performed.

L929 murine fibroblasts (P8) were cultured in DMEM enriched with 10% foetal bovine serum, 1% l-Glutamine (200 millimolar), and 1% Penicillin-Streptomycin in a 5% CO^2 atmosphere. Cells were seeded into the 24-well plate in the presence of the hydrogel cell media at a density of 0.05×10^6 cells per construct, supplied with 1 mL of cell culture medium and incubated overnight. The medium was changed every 2 to 3 days, and samples were grown for a duration of up to 7 days. For the assessment of cytotoxicity, AB was

introduced at 10% of the well volume and incubated for 5 hours. A volume of 100 µl from each well was then transferred in triplicate to a 96-well plate to measure AB cell metabolic activity. Fluorescent emission from the cells was conducted using the SynergyMxBioTek (Bernal Institute, University of Limerick). Cells cultured in the absence of hydrogels served as controls [13].

Following the same method described above, the biocompatibility of the samples was evaluated using Live/Dead staining with Calcein AM and PI. Cells were scanned using the Molecular Devices ImageXpress Micro Confocal (Bernal Institute, University of Limerick) at a wavelength of 488 nm for FITC and AF 594 for Texas Red for fluorescence imaging. The cell viability was quantitatively analysed using ImageJ software in triplicates for each well using Equation 15[13].

$$\%\ Cell\ Viability = \left(\frac{\#\ live\ cells}{\#\ live + dead\ cells}\right) x100$$

Equation 15

1.1.4.1. Statistical analysis

The experiments were conducted in triplicate, and the data is shown as mean ± standard deviation. A two-way ANOVA and Tukey's post-hoc test was used for the AB cytocompatibility assessment. In Figures, single and double asterisks were used to nominate statistical significances with a p-value of < 0.05 being statistically significant (* $p < 0.05$) (95% confidence interval) and < 0.01 being highly significant (** $p < 0.01$) (99% confidence interval). Statistical analysis performed using GraphPad Prism software (La Jolla, CA).

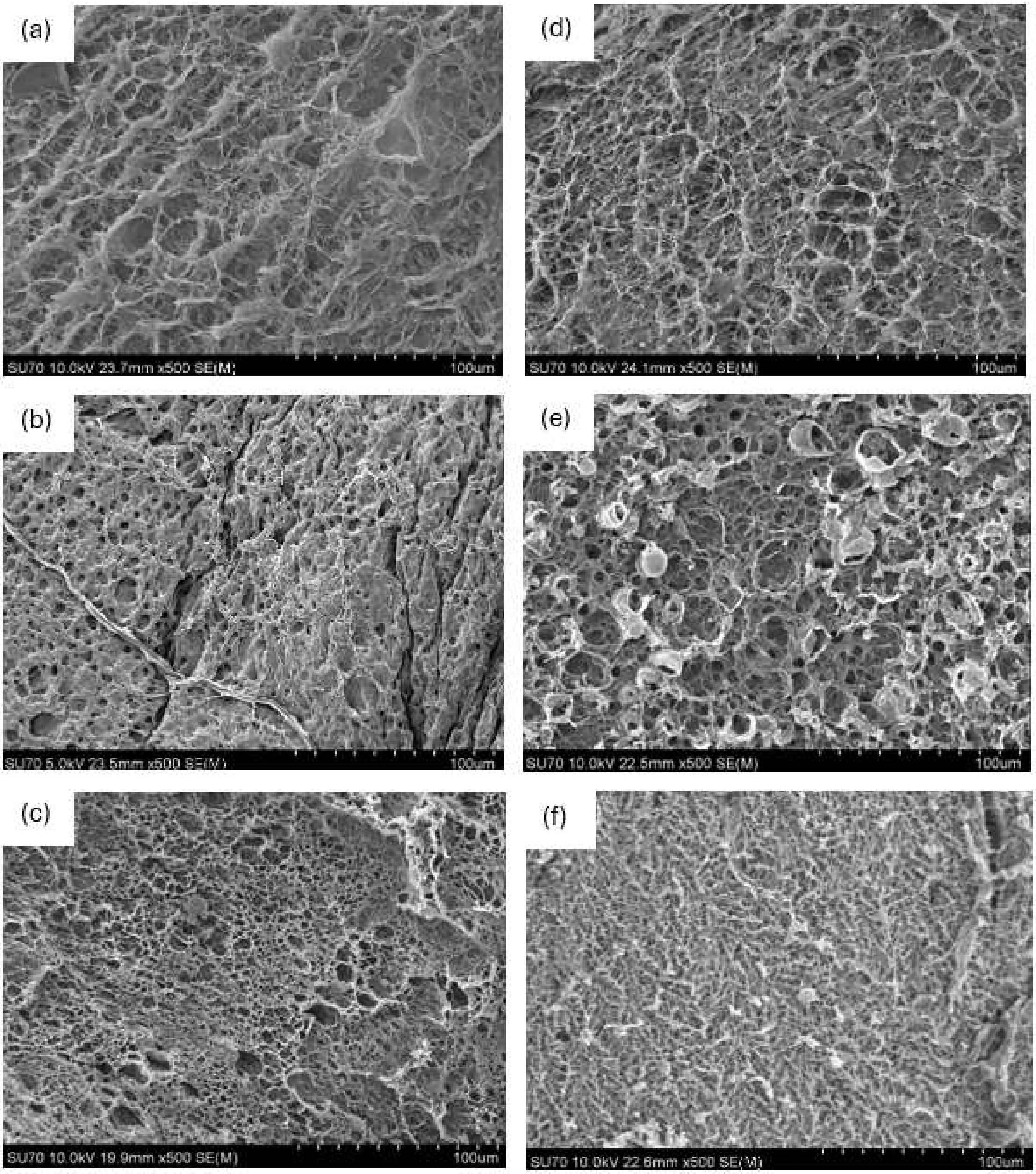

Figure S1: SEM images of lignin-PVA hydrogels with different crosslinker concentrations with (a) K-2.5%, (b) K-5%, (c) K-10%, (d) O-2.5%, (e) O-5%, and (f) O-10%. All images taken at x300 and 10kV at top view.

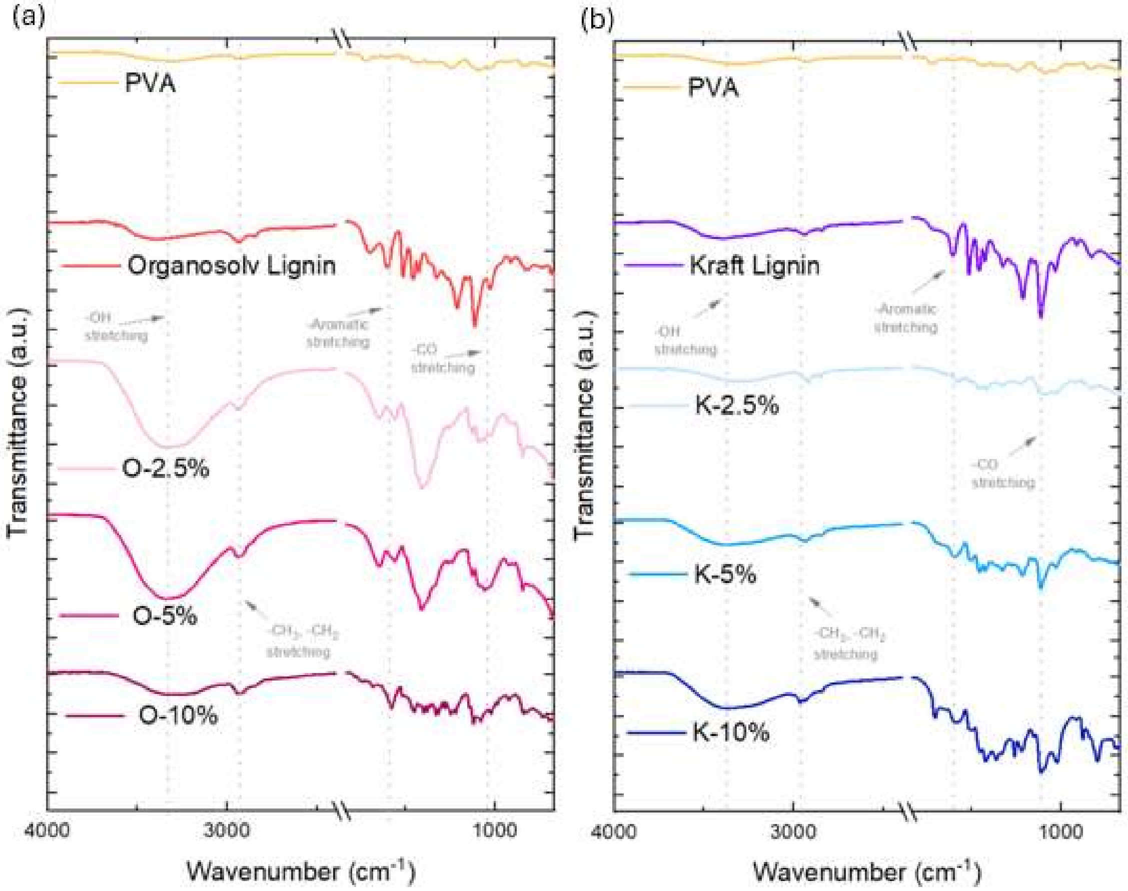

Figure S2: FTIR spectra of the hydrogels with different crosslinker concentrations and their raw component materials where; a) Organosolv lignin and b) Kraft lignin

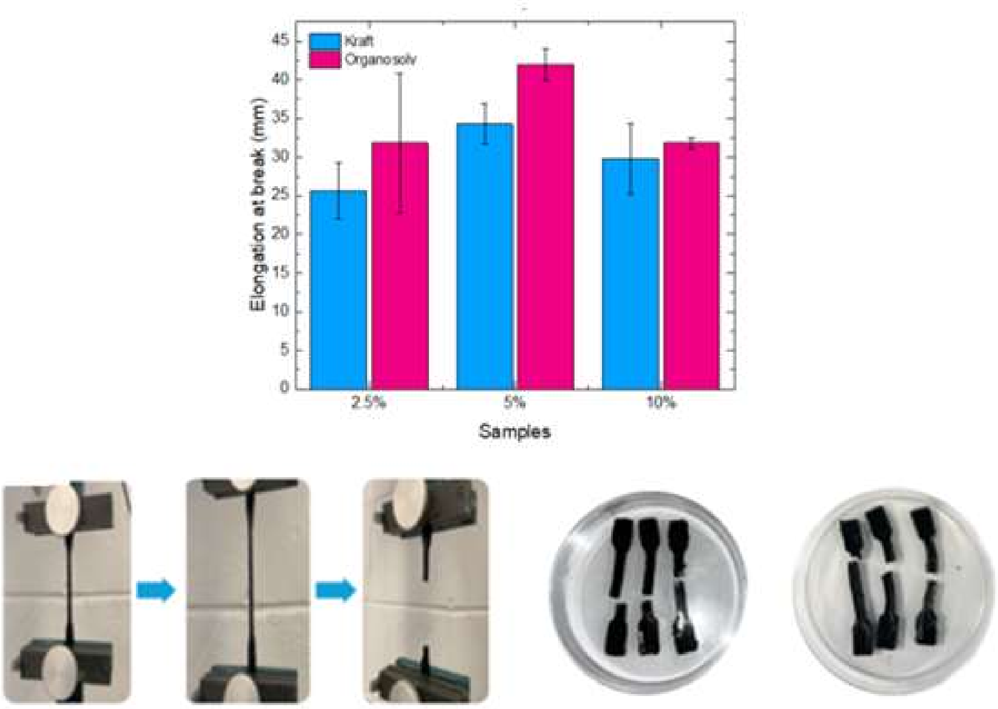

Figure S3: Elongation at break data from uniaxial tensile testing of hydrogels. Representative digital images (from left to right) show K-5% during testing, K-5% after testing, and O-5% after testing. All mechanical tests were performed using a 200 N load cell at a crosshead speed of 10 mm/min (n = 3, mean ± SD).

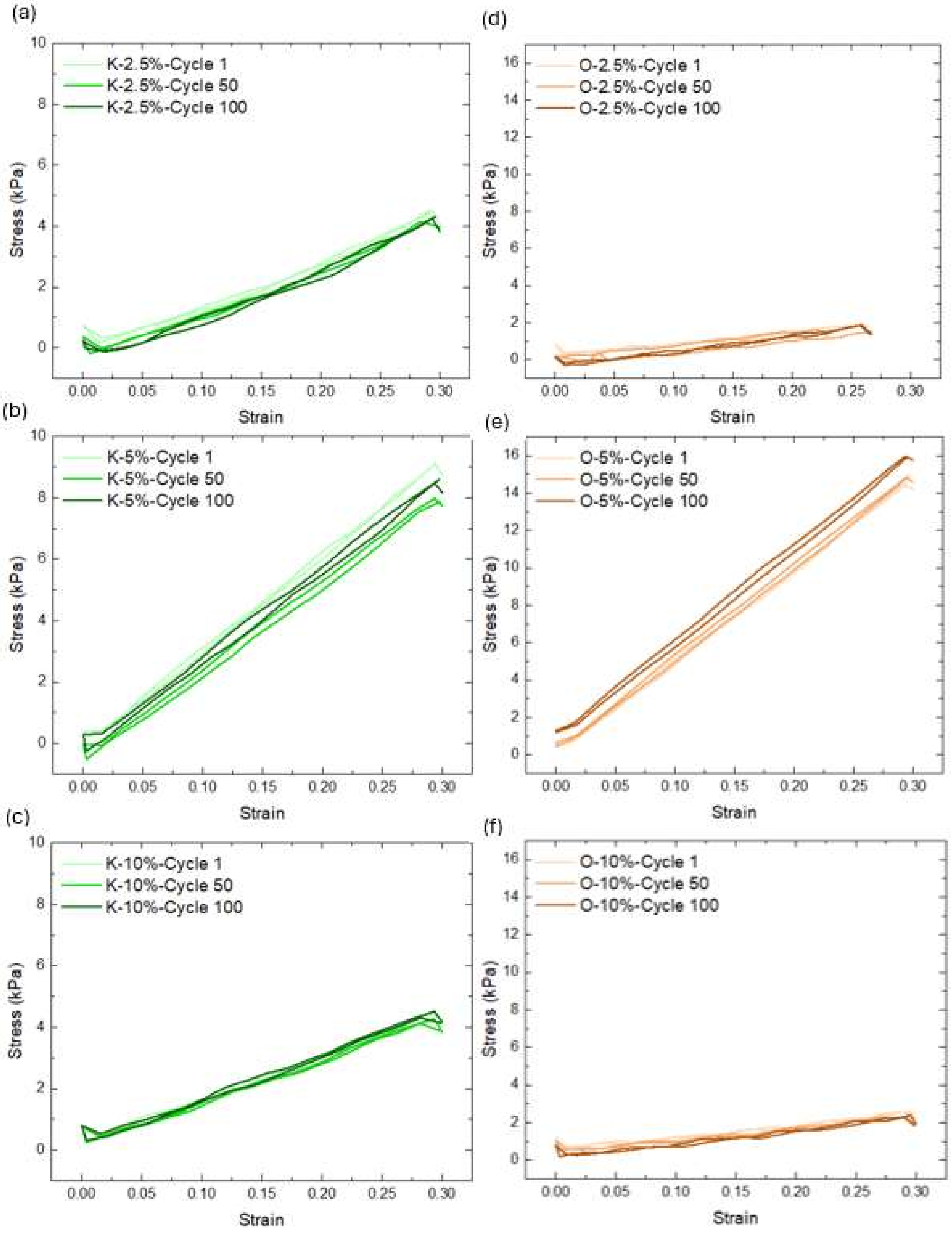

Figure S4. Tensile fatigue cycling results 100 Cycle Fatigue tests in tension of (a) K-2.5% (b) K-5% (c) K-10% (d) O-2.5% (e) O-5% (f) O-10%. All tests performed with a 200N load cell at a rate of 50 mm/min (n=1)

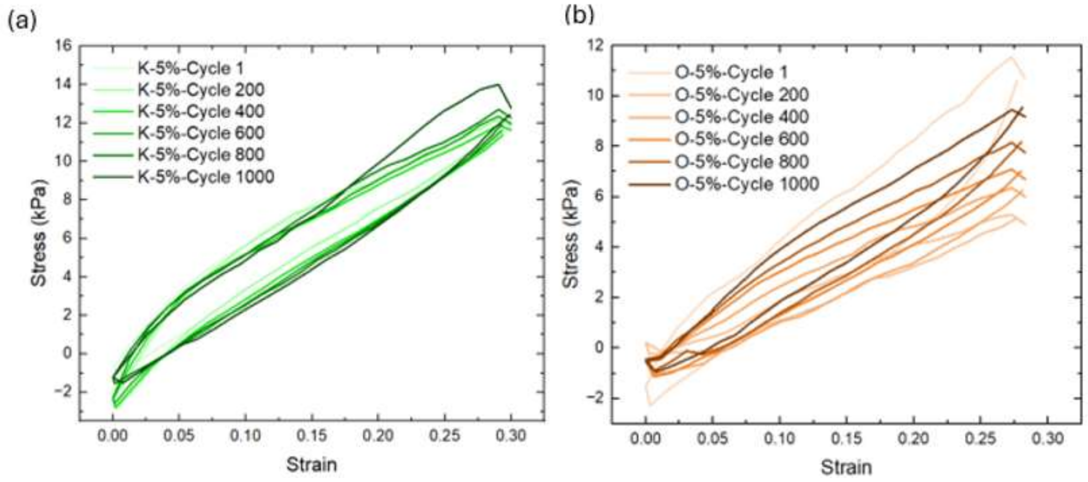

Figure S5. Tensile fatigue cycling results of K-5% and O-5% hydrogels (up to 1,000 cycles), used to assess mechanical durability as a preliminary evaluation of degradation-related performance under repeated loading.

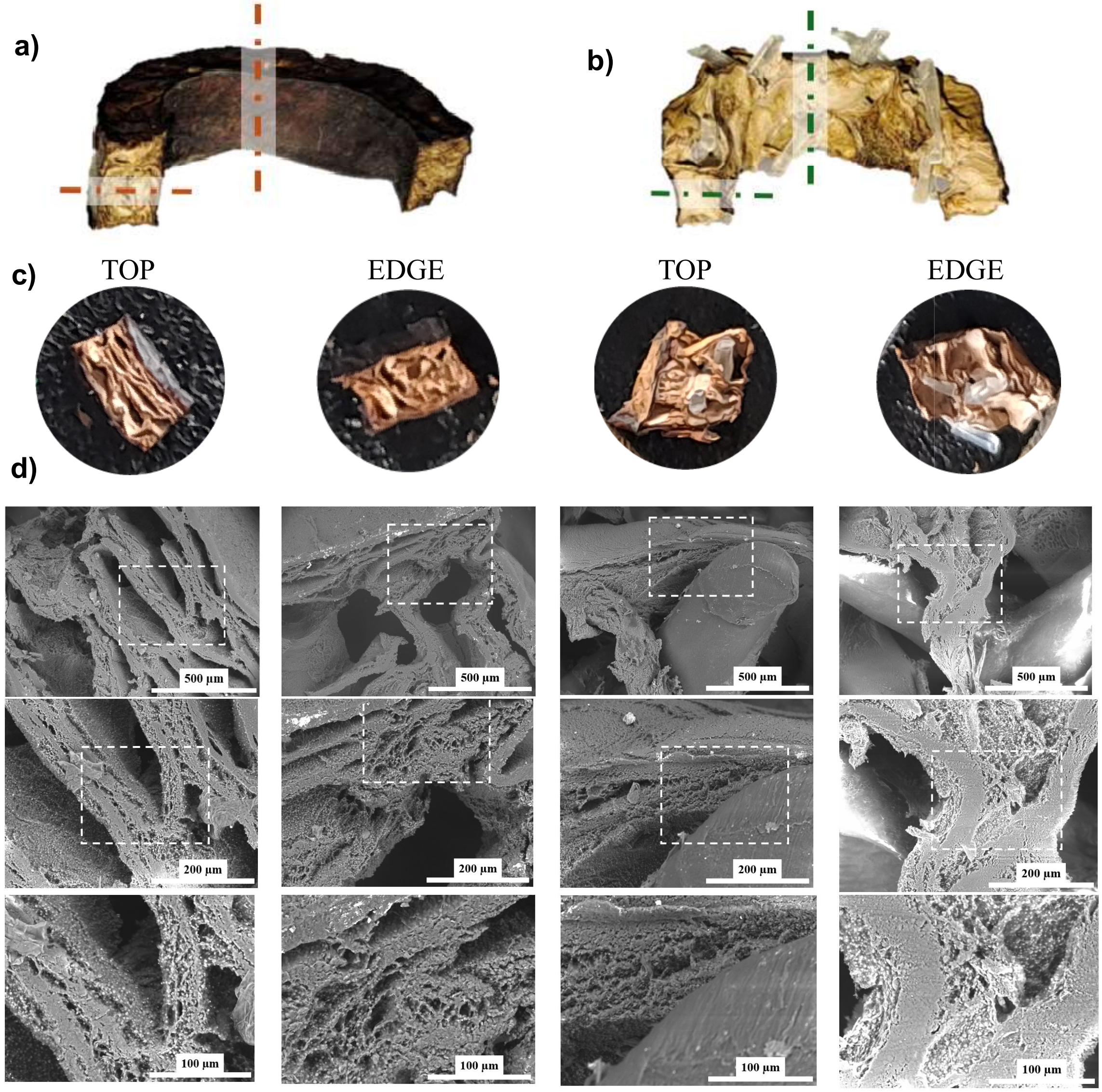

Figure S6. Morphology of the two constructs. a) Digital images of the hydrogels NoR-t3 and b) R-pp-t3 with **c)** their respective top and edge cross-sections. **d)** SEM pictures at microscale of each cross-section sample with magnification of x200, x500 and x1000.

Table S1: Tensile Fatigue Analysis – Young's Modulus of Hydrogel Samples at Cycle 1 and Cycle 100

Young's Modulus (kPa)

Sample	Cycle 1	Cycle 100
K-2.5%	13.7295	9.918202
K-5%	27.33734	28.49458
K-10%	16.90137	16.54682
O-2.5%	9.003619	5.978899
O-5%	49.2413	61.23272
O-10%	12.93728	9.705925

Table S2: Tensile Fatigue Analysis – Young's Modulus of Hydrogel Samples at Cycle 1-Cycle 1000

Young's Modulus (kPa)

Cycle Number	K-5%	O-5%
Cycle 1	43.40625	31.61934
Cycle 200	43.15341	16.10499
Cycle 400	40.26778	21.82527
Cycle 600	39.55845	22.58538
Cycle 800	39.24633	26.23919
Cycle 1000	38.02849	29.96811

References

[1] T. M. G. Selva, J. S. G. Selva, and R. B. Prata, "Sensing Materials: Diamond-Based Materials," in *Encyclopedia of Sensors and Biosensors (First Edition)*, R. Narayan Ed. Oxford: Elsevier, 2023, pp. 45-72.

[2] A. Nanakoudis. "What is SEM? Scanning Electron Microscopy Explained." ThermoFisher Scientific. https://www.thermofisher.com/blog/materials/what-is-sem-scanning-electron-microscopy-explained/ (accessed.

[3] M. Fodil Cherif, D. Trache, N. Brosse, F. Benaliouche, and A. Tarchoun, "Comparison of the Physicochemical Properties and Thermal Stability of Organosolv and Kraft Lignins from Hardwood and Softwood Biomass for Their Potential Valorization," *Waste and Biomass Valorization,* 12/01 2020, doi: 10.1007/s12649-020-00955-0.

[4] S. Obeso Diaz and A. Viplav. "*Connection: OD, Absorbance, and Transmittance in Spectrophotometry*." https://byonoy.com/journal/understanding-od-absorbance-transmittance-spectrophotometry/ (accessed.

[5] A. Morales, J. Labidi, and P. Gullón, "Assessment of green approaches for the synthesis of physically crosslinked lignin hydrogels," *Journal of Industrial and Engineering Chemistry,* vol. 81, pp. 475-487, 2020/01/25/ 2020, doi: https://doi.org/10.1016/j.jiec.2019.09.037.

[6] H. M. Ngwangwa and F. Nemavhola, "Evaluating computational performances of hyperelastic models onsupraspinatus tendon uniaxial tensile test data," *Journal of Computational Applied Mechanic,* p. 17, 2021, doi: 10.22059/jcamech.2020.310491.559.

[7] W. Nafo and A. Al-Mayah, "Characterization of PVA hydrogels' hyperelastic properties by uniaxial tension and cavity expansion tests," *International Journal of Non-Linear Mechanics,* vol. 124, p. 103515, 2020/09/01/ 2020, doi: https://doi.org/10.1016/j.ijnonlinmec.2020.103515.

[8] Á. Pérez-Benito, C. Huerta-López, J. Alegre-Cebollada, J. M. Garcia Aznar, and S. Hervas-Raluy, "Computational modelling of the mechanical behaviour of protein-based hydrogels," *Journal of the Mechanical Behavior of Biomedical Materials,* vol. 138, p. 105661, 02/01 2023, doi: 10.1016/j.jmbbm.2023.105661.

[9] Z. R. Sitte, T. S. Larson, J. C. McIntosh, M. Sinanian, and M. R. Lockett, "Selecting the appropriate indirect viability assay for 3D paper-based cultures: a data-driven study," (in eng), *Analyst,* vol. 148, no. 10, pp. 2245-2255, May 16 2023, doi: 10.1039/d3an00283g.

[10] E. M. Longhin, N. El Yamani, E. Rundén-Pran, and M. Dusinska, "The alamar blue assay in the context of safety testing of nanomaterials," (in eng), *Front Toxicol,* vol. 4, p. 981701, 2022, doi: 10.3389/ftox.2022.981701.

[11] L. Khalef, R. Lydia, K. Filicia, and B. Moussa, "Cell viability and cytotoxicity assays: Biochemical elements and cellular compartments," *Cell Biochemistry and Function,* vol. 42, no. 3, p. e4007, 2024/04/01 2024, doi: https://doi.org/10.1002/cbf.4007.

[12] A. Serafin, M. Culebras, J. M. Oliveira, J. Koffler, and M. N. Collins, "3D printable electroconductive gelatin-hyaluronic acid materials containing polypyrrole nanoparticles for electroactive tissue engineering," *Advanced Composites and Hybrid Materials,* vol. 6, no. 3, p. 109, 2023/05/20 2023, doi: 10.1007/s42114-023-00665-w.

[13] A. Serafin, "Bio-inspired electroconductive scaffolds for spinal cord injury repair," Doctoral, Faculty of Science and Engineering, University of Limerick 2023. [Online]. Available: https://researchrepository.ul.ie/articles/thesis/Bio-inspired_electroconductive_scaffolds_for_spinal_cord_injury_repair/24087810/1?file=42270465
